# Investigating the individual and combined effects of coenzyme Q10 and vitamin C on CLP-induced cardiac injury in rats

**DOI:** 10.1038/s41598-024-52932-5

**Published:** 2024-02-07

**Authors:** Hilal Üstündağ, Özlem Demir, Mehmet Tahir Huyut, Neslihan Yüce

**Affiliations:** 1 Department of Physiology, Faculty of Medicine, Erzincan Binali Yıldırım University, Erzincan, Türkiye; 2 Department of Histology, Faculty of Medicine, Erzincan Binali Yıldırım University, Erzincan, Türkiye; 3 Department of Biostatistics, Faculty of Medicine, Erzincan Binali Yıldırım University, Erzincan, Türkiye; 4 Department of Biochemistry, Faculty of Medicine, Atatürk University, Erzurum, Türkiye

**Keywords:** Biochemistry, Physiology, Biomarkers, Cardiology, Health care

## Abstract

Sepsis-induced cardiac injury represents a major clinical challenge, amplifying the urgency for effective therapeutic interventions. This study aimed to delve into the individual and combined prophylactic effects of Vitamin C (Vit C) and Coenzyme Q10 (CoQ10) against inflammatory heart injury in a cecal ligation and puncture (CLP) induced polymicrobial sepsis rat model. Thirty adult female Sprague–Dawley rats were randomly divided into five groups: Control, CLP, Vitamin C, CoQ10, and Vit C + CoQ10, each consisting of six rats. Treatments were administered orally via gavage for 10 days prior to the operation. Eighteen hours post-sepsis induction, the animals were euthanized, and specimens were collected for analysis. The study examined variations in oxidative (TOS, OSI, MDA, MPO) and antioxidative markers (TAS, SOD, CAT, GSH), histopathological changes, inflammatory cytokine concentrations (TNF-α, IL-1β), nitric oxide (NO) dynamics, and cardiac indicators such as CK-MB. Impressively, the combined regimen markedly diminished oxidative stress, and antioxidative parameters reflected notable enhancements. Elevated NO levels, a central player in sepsis-driven inflammatory cascades, were effectively tempered by our intervention. Histological examinations corroborated the biochemical data, revealing diminished cardiac tissue damage in treated subjects. Furthermore, a marked suppression in pro-inflammatory cytokines was discerned, solidifying the therapeutic potential of our intervention. Interestingly, in certain evaluations, CoQ10 exhibited superior benefits over Vit C. Collectively, these findings underscore the potential therapeutic promise of Vit C and CoQ10 combination against septic cardiac injuries in rats.

## Introduction

Sepsis is a multifaceted and complex syndrome characterized by a systemic and uncontrolled response to an infection, frequently culminating in multiple organ dysfunctions and potentially leading to organ failure^1^. Among the affected organs, the cardiovascular system is subjected to severe stress during sepsis, often culminating in what is referred to as septic cardiomyopathy, a finding well-supported by pre-clinical research^2,3^. This cardiac dysfunction, resulting in both direct and indirect damage to the heart, is a multifactorial process, stemming from various pathophysiological mechanisms that operate during sepsis, as demonstrated in both animal models and human studies^4,5^.

The intricate pathophysiological link between sepsis and cardiac injury is routed through several mechanisms, with the immense inflammatory response being a key player. Sepsis induces the release of a vast array of inflammatory cytokines, including but not limited to, tumor necrosis factor-alpha (TNF-α) and interleukin-1 (IL-1)^6^. These inflammatory mediators, often referred to as 'cytokine storm', bear direct cardiotoxic effects. TNF-α, for instance, leads to impaired myocardial contractility by inhibiting beta-adrenergic receptor signaling^7^. Additionally, it can induce apoptosis in cardiomyocytes, leading to the loss of functional myocardium^8^. Interleukin-1, on the other hand, enhances the production of nitric oxide in the myocardium, which can have negative inotropic effects, thereby reducing the contractile capacity of the heart^9^. These inflammatory mediators not only disrupt the normal myocardial architecture and function but also perpetuate a vicious cycle of inflammation, contributing to further cardiac injury and dysfunction.

Continuing from the direct cardiotoxic effects of the inflammatory mediators, another intricate aspect of sepsis pathophysiology revolves around the redox imbalance it induces. An exacerbated systemic inflammatory response stimulates an overproduction of reactive oxygen species (ROS), thereby causing a shift in the body's redox state and leading to oxidative stress. This phenomenon, which harms various cellular structures and catalyzes inflammatory pathways, exacerbating tissue injury and organ dysfunction^10,11^. Concurrently, pro-inflammatory mediators like myeloperoxidase (MPO) are released^12^. The antioxidant system, encompassing elements like catalase (CAT) and glutathione (GSH), endeavors to neutralize the damaging ROS but often gets overwhelmed in the process of sepsis, aggravating tissue damage and propelling the inflammatory response^13^. This oxidative stress, synergized with sepsis-induced metabolic alterations, precipitates a decline in myocardial contractility and overall cardiac output, escalating the risk of heart failure^14^. The systemic vasodilation instigated by sepsis further decreases systemic vascular resistance and cardiac output^15^, thereby accentuating the intertwined connection between sepsis and cardiac dysfunction, necessitating the development of efficacious therapeutic strategies.

The quest for effective therapeutic strategies highlights several potential agents, including CoQ10. This essential mitochondrial cofactor aids in energy production and also functions as a powerful antioxidant. Its role in scavenging free radicals, preventing lipid peroxidation, and preserving mitochondrial function makes it potentially beneficial in mitigating oxidative stress and inflammatory responses seen in sepsis, has been well-documented in both pre-clinical and clinical studies^16^. CoQ10 has been shown to improve cardiac function in conditions of stress and ischemia by enhancing bioenergetics and reducing oxidative damage, a conclusion supported by clinical trials^17,18^.

Similarly, ascorbic acid or Vit C, another potential therapeutic agent, is a potent antioxidant known for its role in neutralizing ROS and reducing oxidative stress. Vit C has been demonstrated to attenuate sepsis-induced damage by enhancing the immune response, improving vasopressor synthesis, and reducing endothelial injury, findings that have been observed in clinical settings^19^. Its role in collagen synthesis also contributes to maintaining the structural integrity of the myocardium and blood vessels, thereby possibly reducing sepsis-associated cardiac injury^20,21^. The potential synergistic action of CoQ10 and Vit C could be a promising avenue to explore in sepsis treatment. As a combination, these agents may offer a more comprehensive approach to modulating the intricate pathophysiological mechanisms underlying sepsis and associated cardiac dysfunction. This potential stems from their joint capacity to scavenge ROS, bolster the antioxidant defense system, and reduce inflammation, thereby potentially improving cardiac function and overall outcomes in sepsis, as evidenced by both pre-clinical and clinical research^5,22,23^.

Previous studies have extensively explored the effects of Vit C and CoQ10 on sepsis-induced cardiac injury^17,18,21,24,25^. However, these investigations predominantly focused on their individual impacts rather than their combined efficacy. Our study introduces a novel perspective by examining the synergistic effects of Vit C and CoQ10 in mitigating cardiac dysfunction in sepsis. We aim to bridge the gap in existing literature by investigating their combined action on oxidative stress, inflammatory responses, and cardiac enzymatic markers in sepsis-induced cardiac injury. This approach is anticipated to uncover novel insights into the potential of these agents as a combined therapy, thereby contributing to the development of more effective therapeutic strategies for sepsis-related cardiac complications. In addition to investigating the combined effects of Vit C and CoQ10, our study assesses which agent is more effective in combating sepsis-induced cardiac dysfunction. This aspect adds a critical dimension to our research, further delineating the therapeutic potential of these antioxidants in sepsis management.

Building upon the demonstrated potential of CoQ10 and Vit C, this study is designed to scrutinize their impacts in the context of sepsis-induced cardiac dysfunction. By evaluating their individual and combined efficacy, we aim to elucidate their role in mitigating oxidative stress, controlling inflammatory responses, and influencing cardiac enzymatic markers. Through this, we aspire to enhance our understanding and potentially guide the development of innovative therapeutic strategies for sepsis.

## Materials and methods

### Animals

The study employed adult female Sprague–Dawley rats, totaling 30 individuals, with each experimental group comprising 6 rats. These rats were sourced from Atatürk University's Experimental Animal Laboratory of Medicinal and Experimental Application and Research Center. All rats were aged between 8 and 10 weeks. The study strictly adhered to the ARRIVE guidelines and was conducted following the national regulations for the ethical use and care of laboratory animals. Ethical approval was obtained from Ataturk University's local animal care committee (Approval date: 2021, Session no: 11, Decision no: 258).

Animals were housed in standard cages with sawdust bedding and maintained under controlled environmental conditions (22 ± 1 °C), with a 14-h light/10-h dark cycle. They had ad libitum access to standard rodent chow and tap water throughout the study.

For surgical interventions and sample collection, rats were anesthetized through intraperitoneal administration of a combined dose of 100 mg/kg ketamine and 15 mg/kg xylazine. Following the completion of the study, the animals were humanely euthanized using cervical dislocation to ensure minimal suffering.

### Experimental design models and groups

**Figure 1:**
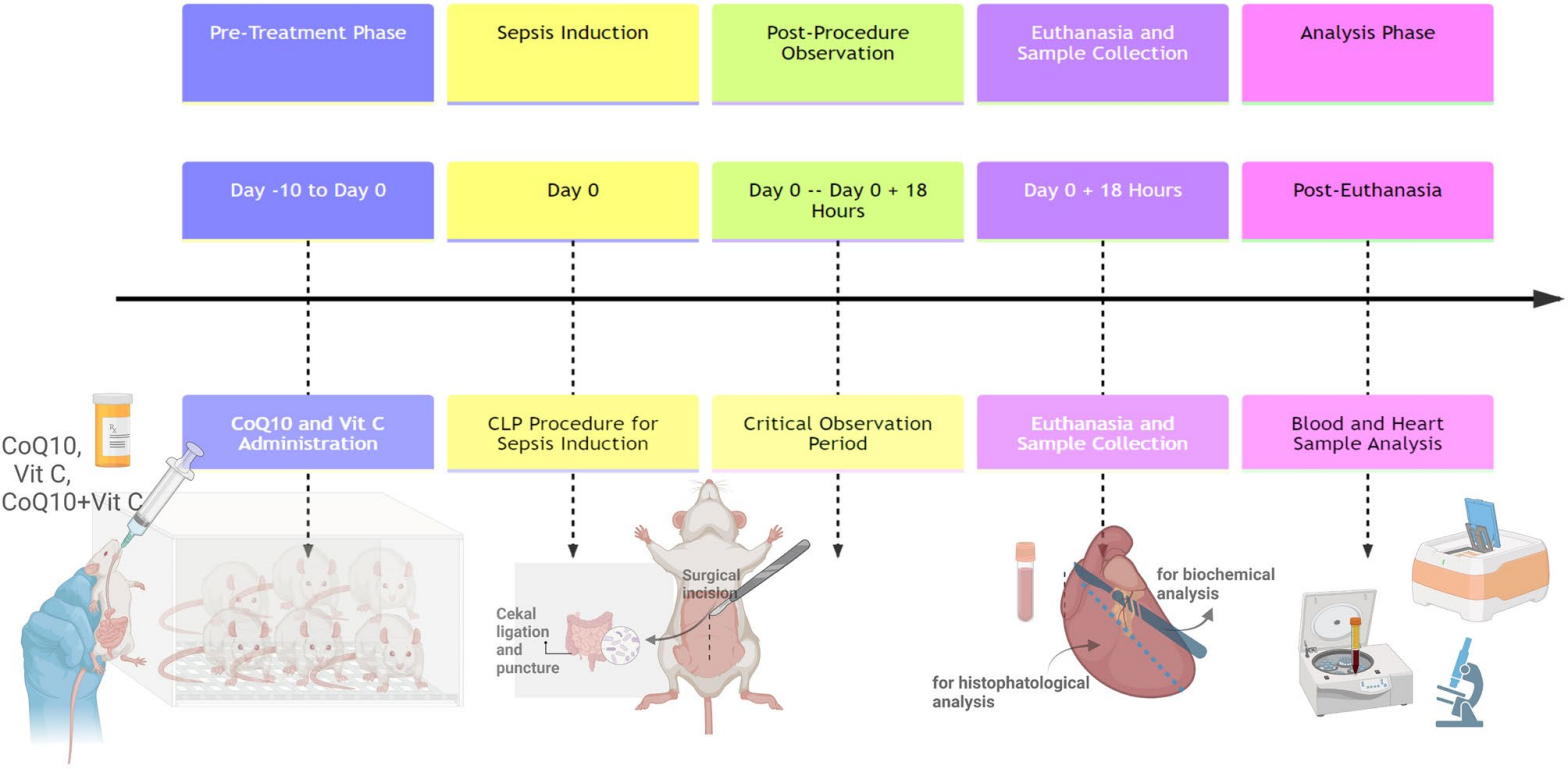
Timeline of the experimental design, illustrating the key phases from pre-treatment to analysis.

In this study, a detailed overview of the entire experimental process is provided to elucidate the treatment and observation timeline (Fig. 1). The experimental design involved oral administration of treatments (gavage) before the induction of sepsis. Specifically, treatments with CoQ10 and Vit C, either individually or in combination, were administered for 10 days preceding the sepsis induction. This pre-treatment was aimed at evaluating the prophylactic effects of these compounds against sepsis-induced cardiac injury.

The sepsis induction was conducted using the CLP procedure, a widely recognized method for creating a polymicrobial sepsis model. Following the CLP procedure, a critical observation period of 18 h was established, after which the animals were euthanized^26^. This timeline was chosen based on previous studies indicating the peak of inflammatory and oxidative stress responses post-sepsis induction within this timeframe^27^.

Our study was divided into five distinct groups, each comprising six rats, and each serving a unique role within the experimental design.

#### Control (sham) group

These rats underwent a sham operation, which involved the same procedure as the cecal ligation and puncture (CLP) operation but without ligation and perforation of the cecum, serving as the baseline control for the other groups. They were neither subjected to the sepsis-inducing CLP procedure nor treated with any therapeutic agents, therefore, representing normal physiological conditions.

#### CLP group

In this group, rats underwent the CLP procedure to induce sepsis but received no subsequent therapeutic interventions. This group served to demonstrate the effects of sepsis in the absence of any mitigating treatments.

#### CLP + CoQ10 group

Rats in this group were pre-treated with CoQ10 at a dose of 300 mg/kg^24^ through gavage for 10 days prior to sepsis induction via the CLP procedure. After the CLP procedure, no further CoQ10 was administered, and the rats were euthanized at the 18th hour.

#### CLP + Vit C group

Before undergoing the CLP-induced sepsis procedure, rats in this group were administered Vit C at a dose of 100 mg/kg^5,28^ for 10 consecutive days using gavage. After the CLP method, no further Vit C was given, and the rats were euthanized 18 h later.

#### CLP + CoQ10 + Vit C group

Rats in this group were given a combined regimen of Coenzyme Q10 and Vit C via gavage for 10 days leading up to the sepsis induction using the CLP method. Post-CLP, no additional agents were provided, and the animals were euthanized on the 18th hour.

### Induction of sepsis via CLP

In this study, a polymicrobial sepsis model was generated utilizing the widely accepted CLP method^29^. The rats underwent a 12-h fast prior to the commencement of the experiment. Following the fasting period, rats were anesthetized using an intraperitoneal administration of a combined dose of 100 mg/kg ketamine and 15 mg/kg xylazine. Post-anaesthesia, the abdominal region of each animal was prepared by shaving and disinfecting the area. A 1 cm median incision was made in the abdominal wall to expose the peritoneum. The cecum was then delicately isolated, taking care not to damage surrounding abdominal organs. Ischemia and circulatory disturbances were induced by ligating the area distal to the ileocecal valve using a 4/0 silk suture. Subsequent to the ligation, an 18-gauge needle was used to create two perforations on the opposite sides of the mesentery, resulting in a total of four holes from the distal of the cecum to the ligation point. By milking the cecum, bacterial flora from the feces were released into the peritoneal cavity, thus inducing abdominal sepsis. The cecum was then carefully placed back into the peritoneal cavity. Prior to closing the abdominal incision, 0.5 mL of normal saline was administered into the peritoneal cavity of each animal to prevent dehydration and to maintain electrolyte balance. The abdominal incision was then securely closed in two layers using the Covidien Appose ULC Auto Suture Slim Body Skin Stapler 35 W (Covidien, India).

Post-sepsis induction, the rats were euthanized using an intraperitoneal injection of ketamine and xylazine. Blood samples were then collected through intracardiac puncture to analyze systemic inflammatory and oxidative markers. Heart was rapidly excised, rinsed with ice-cold saline to remove blood contaminants, and processed for subsequent analysis. One half of the heart was preserved at − 80 °C for biochemical assays, while the other half was fixed in 10% formalin for detailed histopathological examination.

### Drug administration

CoQ10 was procured from Solgar Inc., and Vit C was obtained from Solarbio Science & Technology Co., Ltd. Both agents were administered to the appropriate groups by oral gavage for ten days preceding the CLP procedure.

CoQ10 is a potent lipophilic antioxidant and a critical co-factor for mitochondrial electron transport. Its administration aimed at boosting the body's antioxidant capacity and mitigating the oxidative stress associated with sepsis-induced cardiac damage. This administration protocol is consistent with prior studies showing the beneficial impact of CoQ10 in different models of animals and humans^30,31^.

Ascorbic Acid, commonly known as Vit C, is a potent antioxidant and a cofactor for various enzymes. Its supplementation aimed to strengthen the antioxidant defense system and reduce oxidative stress and inflammation during sepsis, thereby potentially mitigating sepsis-induced cardiac damage^32^. The duration of Vit C administration and its oral gavage administration route aligns with previous studies demonstrating its efficacy in alleviating sepsis-induced complications^25^.

This pre-emptive administration strategy aimed to establish an optimal therapeutic concentration of both agents in the body prior to sepsis induction, thereby potentially maximizing their therapeutic benefits in the context of sepsis-induced cardiac damage.

### Biochemical investigations

#### Tissue homogenization

In the study, blood samples were first collected from the euthanized rats' hearts via intracardiac puncture. These samples were then centrifuged at 3500×*g* for 10 min to obtain the serum, which was subsequently examined for biochemical parameters. Following the collection of blood samples, tissue homogenization was carried out. In this process, a KH_2_PO_4_/K_2_HPO_4_ buffer solution (50 mM, pH 7.2) was utilized. A 100 mg tissue sample was dissected and placed in 1 mL of the buffer solution. The tissue samples were then homogenized using a homogenizer at 5000 rpm within an ice bath. After homogenization, the samples were transferred to eppendorf tubes and centrifuged at 1600×*g*, at 4 °C for 20 min. The supernatants were used for biochemical parameter measurements.

### Biochemical parameter measurement

Frozen samples were gradually thawed on the day of analysis. Multiple freeze–thaw cycles were avoided. All standards, controls, kits, and samples were equilibrated to room temperature (18–26 °C) prior to use.

#### Tissue Protein Measurement

Heart tissue protein concentrations were quantified using the Comassie Blue G-250 protein assay, commonly known as the Bradford method^33^. This colorimetric technique involves the binding of the dye to proteins, resulting in a measurable color change proportional to the protein concentration. The results were accurately calculated using a linear calibration curve, which was established with albumin standards. These standards ranged from 15.62 to 1000 µg/mL, ensuring a broad and precise measurement range for the accurate quantification of protein levels in the heart tissue samples.

#### TAS, TOS and OSI measurements

##### TAS measurement

TAS level in tissue samples were measured by Erel method^34^. The total antioxidant capacity in heart tissue samples was quantified utilizing a commercial kit from Rel Assay Diagnostics (Catalog no: RL0017, Gaziantep, Türkiye). The assay principle involves the reduction of the ABTS radical, which is dark blue-green in color, to a colorless reduced form by the antioxidants in the sample. The change in absorbance at 660 nm, which is measurable spectrophotometrically, correlates with the total antioxidant level in the sample. Calibration of this test was done using a balanced antioxidant standard solution, known as Trolox Equivalent. The results were calculated using a 1 mmol Trolox Equivalent per Liter standard, with the manufacturer reporting a Coefficient of Variation (CV) of ± 10% within the 1.20–1.50 mmol/L range. Results were expressed in mmol/L for tissue samples.

##### TOS measurement

Total oxidant levels in serum samples were measured using a commercial kit from Rel Assay Diagnostics (Catalog no: RL0024, Gaziantep, Türkiye). The assay, developed by Erel, employs an automated measurement method where oxidants in the sample oxidize the iron ion-o-dianisidine complex to ferric ion. This oxidation reaction is enhanced by the abundant glycerol molecules in the reaction medium. In an acidic environment, the iron ion forms a colored complex with xylenol orange. The intensity of the color, measurable spectrophotometrically, reflects the total amount of oxidant molecules in the sample. The CV values for this test, as reported by the manufacturer, are ± 10%, with a range of 4–6 µmol/L. Analysis was calibrated with hydrogen peroxide, and results were presented in µmol H_2_O_2_ Eq./L for tissue samples^35^.

##### OSI

The Oxidative Stress Index was calculated by dividing the Total Oxidant Level by the Total Antioxidant Capacity, obtained using the Erel method. For OSI calculation, the unit of TAS was converted to µmol/L, and the formula TOS (µmol H_2_O_2_ Eq/L)/TAS (µmol/Trolox Eq/L) * 100 was used.

#### Other antioxidant and oxidative stress parameters

##### CAT activity

CAT activity was determined using a spectrophotometric method based on the protocol described by Campo et al. This assay is based on the reduction of absorbance at 405 nm due to the enzymatic degradation of hydrogen peroxide (H_2_O_2_) by catalase. In brief, the process involved incubating 20 µL of supernatant with 100 µL of a substrate solution (composed of 65 mmol/L hydrogen peroxide in a 60 mmol/L phosphate buffer, pH 7.4) at 37 °C for 1 min. The reaction was then stopped by adding 100 µL of ammonium molybdate (32.4 mmol/L), and the absorbance of the resulting solution was measured at 405 nm^36^. Standard solutions were prepared from pure catalase enzyme at different concentrations (156.25-20000 U/mL). Calibration curve was drawn according to the absorbance values of the standards and catalase activity of tissue samples was determined.

##### GPx activity

GPx activity in the samples was determined using a spectrophotometric method, as outlined by Beutler. This assay quantifies GPx activity by monitoring the change in absorbance due to the oxidation of NADPH to NADP+. Specifically, the decrease in absorbance at 340 nm, associated with the consumption of NADPH, is measured to assess the enzymatic activity of GPx. In brief, the assay involves adding a reaction mixture to the sample, which contains the substrate NADPH. The enzymatic activity of GPx in the sample leads to the oxidation of NADPH to NADP+, resulting in a change in absorbance at 340 nm. This change is continuously monitored spectrophotometrically. The rate of decrease in absorbance, which correlates with the rate of NADPH oxidation, is used to calculate the GPx activity in the sample^37^.

##### MDA measurement

MDA levels were quantified by measuring the absorbance of the pink-red color that results from the reaction between MDA and thiobarbituric acid. This method, as outlined by Ohkawa et al. is a well-established spectrophotometric technique for assessing lipid peroxidation products^38^. The pink colored complex formed by the reaction is measured at a wavelength of 532 nm following a 60-min incubation at 95 °C. To ensure accuracy, a stock standard solution of 1,1,3,3-tetraethoxypropane with a concentration of 200 µmol/L was prepared. From this stock, serial dilutions were made to obtain standard solutions at various concentrations. These solutions were used as standards in our assay, and the results were expressed in micromolar (µM) units, providing a quantitative assessment of MDA levels indicative of lipid peroxidation in the tissue samples.

##### MPO activity

MPO activity was determined by measuring the kinetic change in absorbance of a yellowish-orange complex. This complex is formed by the oxidation of o-dianiside in the presence of MPO and hydrogen peroxide, as described in the method by Bradley et al. The assay specifically measures the change in absorbance at a wavelength of 460 nm, which is indicative of MPO enzymatic activity. In this process, a reaction mixture containing o-dianiside and hydrogen peroxide is added to the sample. MPO present in the sample catalyzes the oxidation of o-dianiside, leading to the formation of a yellowish-orange colored complex. The rate of formation of this complex, reflected by the increase in absorbance at 460 nm, is directly proportional to the activity of MPO in the sample^39^.

#### Inflammatory cytokines

##### IL-1β levels

IL-1β levels in the heart tissue were measured using a commercial enzyme-linked immunosorbent assay (ELISA) kit (Bioassay Technology Laboratory, Catalog No: E0092Ra, Zhejiang, China). This method is based on the ELISA principle where the IL-1β in samples is sandwiched between the pre-coated IL-1β antibody on the plate and the biotinylated IL-1β detection antibody added during the assay. The biotinylated detection antibody is then recognized by streptavidin-HRP, forming an immune complex. After subsequent washing steps, the substrate solutions are added, causing a colorimetric reaction which is proportional to the amount of IL-1β in the samples.

##### TNF-α levels

TNF-α levels in the heart tissue were assessed using a commercial kit (Bioassay Technology Laboratory, Catalog No: E0764Ra, Zhejiang, China). The manufacturer reported the CV% values as <10%, with the test's sensitivity pegged at 2.51 ng/L and a range between 5 and 1000 ng/L. Standard solutions ranging from 40 to 640 ng/L were prepared, and results were deduced using the resulting calibration curve. Outcomes were expressed in ng/L.

#### Evaluation of nitrate and nitrite concentrations

Given the ephemeral nature of NO, it rapidly interacts with various molecules found within biological systems. This leads to a cascade of reactions, summarized as follows:

- 2NO + O_2_ → 2NO_2_ which equilibrates with N_2_O_4_.
- N_2_O_4_ + 2OH^−^ yields ${\text{NO}}_{2}^{ - }$ + ${\text{NO}}_{3}^{ - }$ and water.
- NO_2_ combines with NO to form N_2_O_3_.
- N_2_O_3_ reacting with 2OH^−^ produces 2${\text{NO}}_{2}^{ - }$ and water.

The terminal derivatives of NO are nitrate (${\text{NO}}_{3}^{ - }$) and nitrite (${\text{NO}}_{2}^{ - }$). Since the relative proportions of NO_2_ and NO_3_ are not consistent and can't be accurately anticipated, the optimal way to gauge the total NO yield is by summing the concentrations of both ${\text{NO}}_{2}^{ - }$ and ${\text{NO}}_{3}^{ - }$.

To quantify NO levels, we employed a standardized colorimetric test kit (Cayman, Nitrate/Nitrite Colorimetric Assay, Item No: 780001). As per the kit's instructions, nitrate (${\text{NO}}_{3}^{ - }$) gets converted to nitrite (${\text{NO}}_{2}^{ - }$) via the action of nitrate reductase, allowing for an aggregate ${\text{NO}}_{2}^{ - }$ analysis. The kit's precision, indicated by the coefficient of variation (CV), is noted as 2.7% for intra-assay and 3.4% for inter-assay discrepancies.

#### Examination of serum myocardial function marker

When evaluating myocardial function markers in serum, CK-MB is a key enzyme to measure. Serum levels of CK-MB were measured using a commercial ELISA kit for rats (Sunred Biological Technology, Rat kit, Catalog no: 201-11-0312, Shanghai, China). The coefficient of variation (CV%) for the test, as reported by the manufacturer, is <10%. The sensitivity of the test is 0.225 U/L with a range of 0.25–720 U/L. Standard solutions ranging from 25 to 400 U/L were prepared, and results were determined using the generated calibration curve. The outcomes are presented in U/L.

### Histological examination

Heart tissue samples from rats were initially fixed in formaldehyde for a period of 72 h. Following fixation, they were thoroughly rinsed under tap water for 24 h. Subsequently, the tissue samples underwent a graded alcohol series for dehydration, being sequentially immersed in 70%, 80%, 90%, and 100% alcohol solutions. Post-dehydration, the samples were cleared in xylene to render them transparent, after which they were embedded in paraffin wax. From these paraffin blocks, sections of 4 µm thickness were cut. These sections were then stained using hematoxylin and eosin (H&E) based on the technique described by Gamble et al.^40^. The prepared cardiac tissue sections were imaged using a microscope equipped with the DP2-SAL software program (Olympus Inc., Tokyo, Japan). For semi-quantitative scoring, six fields from the serial sections were chosen. In each selected field, degeneration criteria were scored for each specimen. The histopathological criteria in the chosen fields for each specimen were scored semi-quantitatively as: 0 for no damage, 1 for mild damage, 2 for moderate damage, and 3 for severe damage.

### Statistical analysis

In this study, the distinction in parameter and biomarker values due to experimental intervention was determined using effect size (Eta-η2). A statistical power analysis revealed an effect size of 0.8, setting alpha at 0.05 and power at 0.83, indicating a required minimum of 30 subjects. The normality assumption of the data was examined with the Shapiro–Wilk test, and the homogeneity of group variances was checked with the Levene test. While the descriptive statistics of the parameters providing the assumption of normal distribution were presented as mean, standard deviation and confidence intervals, otherwise the median and interquartile range values of the variables were given. Differences between control and experimental groups for certain biomarkers were identified using one-way ANOVA analysis. Then, Tukey HSD post-hoc test was used when the assumption of equality of population variances of the groups was provided, otherwise the Games-Howell post-hoc test was used. Histopathology results were converted to semi-quantitative data and analyzed. If the hypotheses of parametric tests for the differences between the histopathology results of the groups were met, one-way ANOVA analysis was used, otherwise Kruskal–Wallis analysis was used. Then, Games–howell or Mann–Whitney U test with Bonferroni correction was used for multiple comparisons. Histograms and box-plot graphs were used to show the distribution of parameters in groups and post-hoc results. All graphs were created at a 95% confidence level, and error bars indicate a width of ± 2 standard deviations. Asymptotic significance values were calculated according to the two-tailed hypothesis of the tests. *p* < 0.05 was considered statistically significant. SPSS (version 26.0, SPSS Inc, Chicago) package program was used for statistical analysis of the data.

## Results

### Oxidative and antioxidative status in cardiac tissue

#### Antioxidant enzyme levels in cardiac tissue

**Figure 2:**
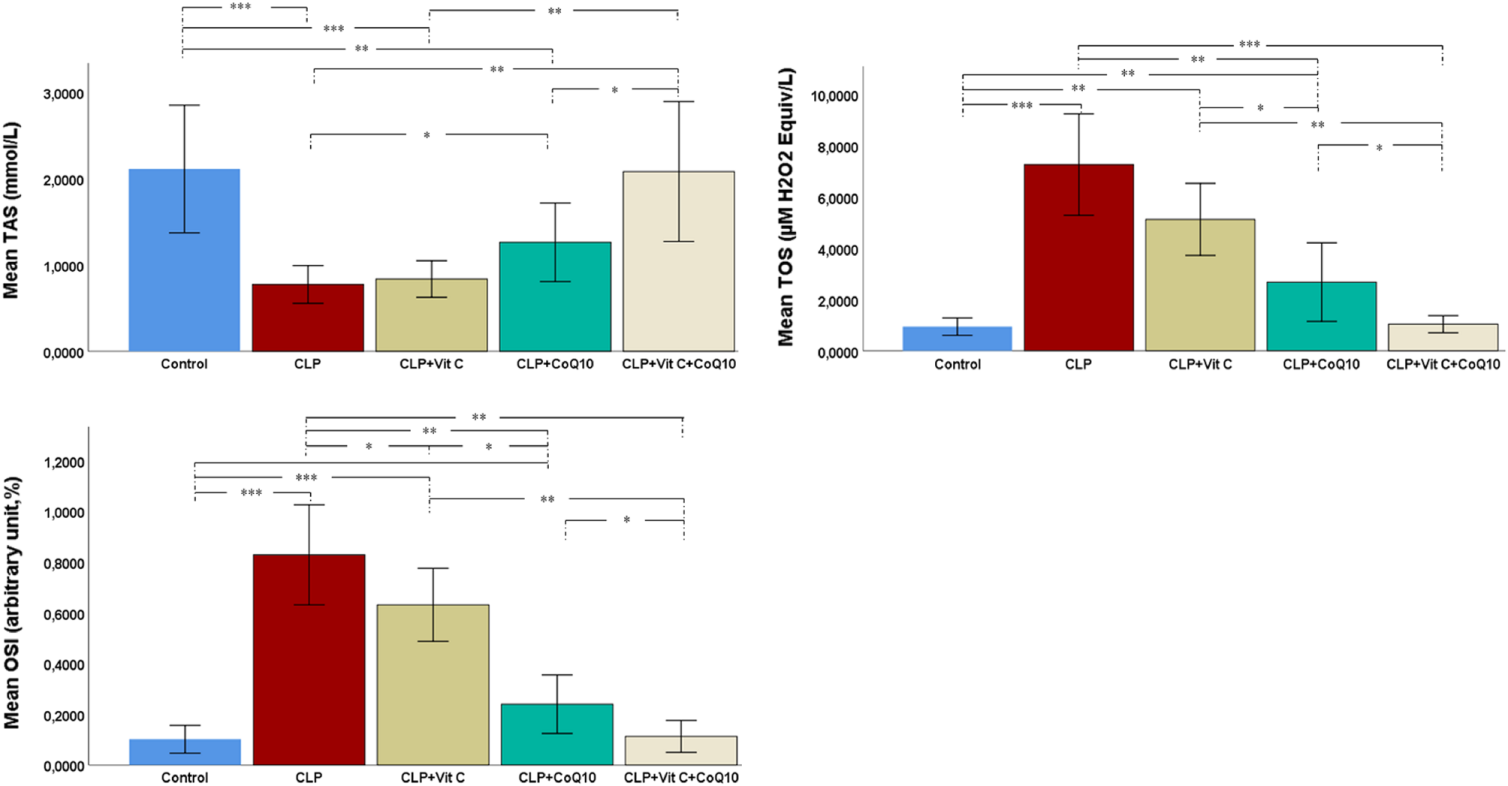
Effects of Vit C, CoQ10 and Vit C + CoQ10 on cardiac TAS, TOS and OSI levels in septic rats (n = 6). Tukey HSD and Games-Howell post-hoc test results used in multiple comparison of group means after one-way ANOVA analysis are shown with *. *p < 0.05, **p < 0.01, ***p < 0.001.

The analysis of antioxidant and oxidant parameters in various experimental groups revealed significant differences in TAS levels (Fig. 2). Notably, the group subjected to CLP-induced sepsis exhibited a marked reduction in TAS compared to the control group, indicating a significant oxidative impact due to sepsis. In contrast, treatments with Vit C and CoQ10 showed a restorative effect on TAS levels, with CoQ10 displaying a slightly more pronounced improvement than Vit C alone. Most notably, the combination of Vit C and CoQ10 effectively restored TAS levels to nearly those observed in the control group.

**Figure 3:**
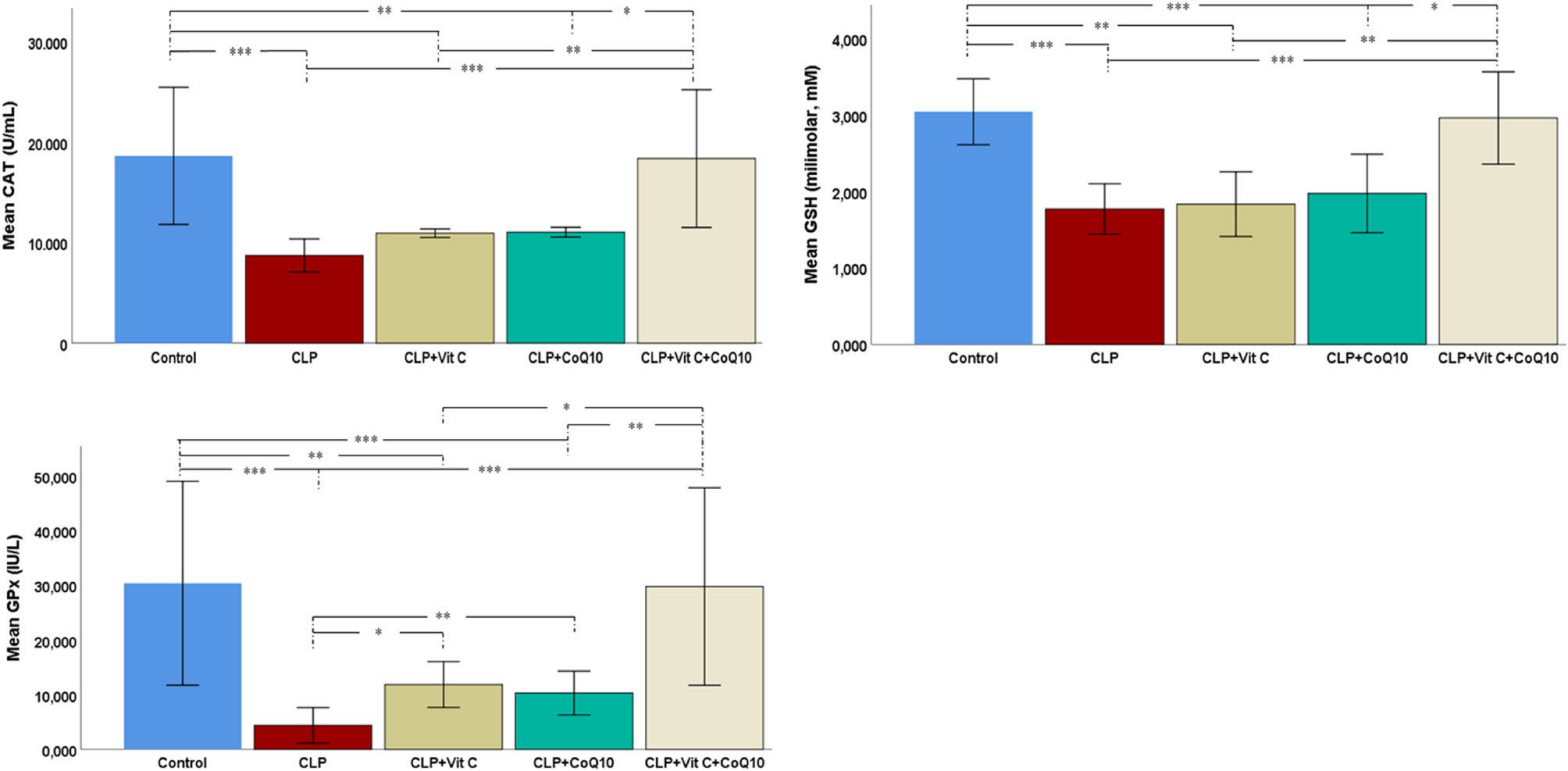
Effects of Vit C, CoQ10 and VitC + CoQ10 on cardiac CAT, GSH and GPx levels in septic rats (n = 6). Tukey HSD and Games-Howell post-hoc test results used in multiple comparison of group means after one-way ANOVA analysis are shown with *. *p < 0.05, **p < 0.01, ***p < 0.001.

Figure 3 presents the antioxidant enzyme levels across the experimental groups. In the group with CLP-induced sepsis, there was a substantial decrease in CAT activity, indicating a significant impact of sepsis on antioxidant enzyme levels, compared to the control group. Treatments with Vit C and CoQ10 individually showed a partial recovery in CAT activity. However, it was the combined treatment of Vit C and CoQ10 that notably restored CAT activity to levels comparable to the control group.

The analysis of GSH levels demonstrated a significant decrease in the CLP group compared to the control group (*p* < 0.001), underscoring the oxidative damage induced by sepsis. Individual treatments with Vit C and CoQ10 led to slight, but statistically significant, improvements in GSH levels (Vit C: *p* < 0.05; CoQ10: *p* < 0.05). However, the combined treatment of Vit C and CoQ10 resulted in a remarkable restoration of GSH levels, closely matching those of the control group (*p* < 0.001).

Regarding GPx activity, a drastic reduction was observed in the CLP group compared to the control (*p* < 0.001). Treatments with Vit C and CoQ10 individually led to significant increases in GPx levels (Vit C: *p* < 0.01; CoQ10: *p* < 0.01). The most substantial improvement was seen with the combined therapy, where GPx levels nearly mirrored those of the control group (*p* < 0.001).

#### Oxidative stress markers in cardiac tissue

**Figure 4:**
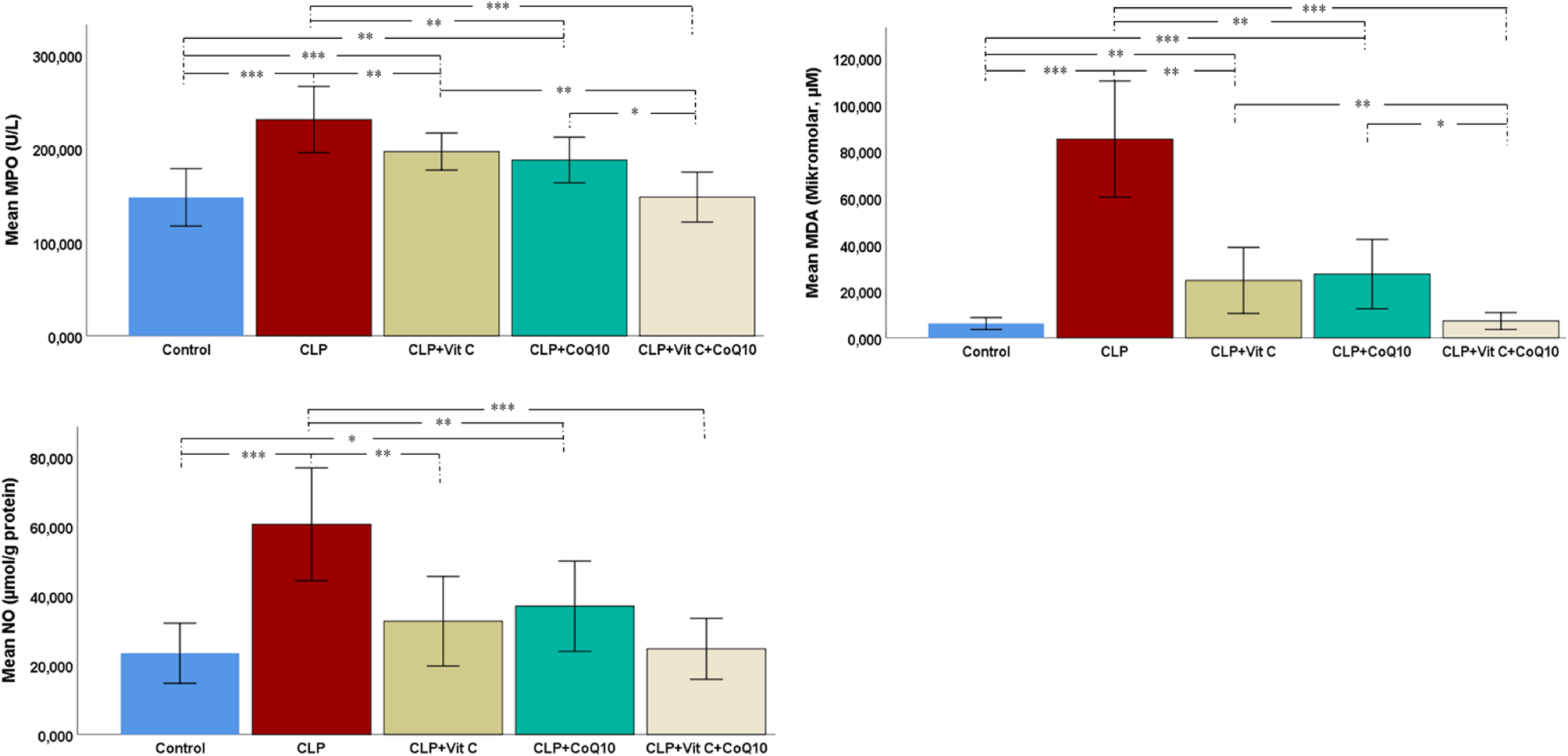
Effects of Vit C, CoQ10 and Vit C + CoQ10 on cardiac MPO, MDA and NO levels in septic rats (n = 6). Tukey HSD and Games-Howell post-hoc test results used in multiple comparison of group means after one-way ANOVA analysis are shown with *. *p < 0.05, **p < 0.01, ***p < 0.001.

From the data in Fig. 4, the influence of treatment on the oxidative stress markers, MPO and MDA, is evident. The data show that the CLP group experienced a marked increase in MPO levels compared to the control group, indicating a substantial elevation of oxidative stress due to sepsis (*p* < 0.001). Treatments with Vit C and CoQ10 individually led to a decrease in MPO levels (*p* < 0.05), with CoQ10 showing a slightly more pronounced reduction. However, it was the combined treatment of Vit C and CoQ10 that had the most significant impact, effectively lowering MPO levels to nearly those of the control group (*p* < 0.001).

Concerning MDA levels, in the CLP group, with values significantly higher than those in the control group (*p* < 0.001). Treatments with Vit C and CoQ10, administered individually, effectively reduced MDA levels, though the combined treatment of Vit C and CoQ10 was notably more effective.

In terms of TOS (Fig. 2), the CLP group exhibited an elevation compared to the control (*p* < 0.001). Both Vit C and CoQ10 treatments were successful in reducing TOS, with CoQ10 showing a more pronounced effect. The combined application of Vit C and CoQ10 resulted in TOS levels nearly equivalent to those in the control group, demonstrating the synergistic potential of this treatment regimen.

The data from our study highlight the regarding the OSI, there was a significant increase in the CLP group in comparison to the control group (*p* < 0.001). Both Vit C and CoQ10 treatments individually showed a capacity to lower OSI, with CoQ10 being more effective than Vit C.

#### Nitric oxide levels in cardiac tissue

Figur 2 highlights the significant alteration in NO levels across different experimental groups (Fig. 4). The CLP group exhibited a marked increase in NO levels compared to the control group, indicating a substantial elevation in response to sepsis (*p* < 0.001). Treatments with Vit C and CoQ10 were effective in reducing these elevated NO levels, with CoQ10 showing a marginally greater efficacy than Vit C. Notably, the combination of Vit C and CoQ10 demonstrated a synergistic effect, bringing NO concentrations down to levels closely aligned with the control group.

#### Inflammatory biomarkers in cardiac tissue

**Figure 5:**
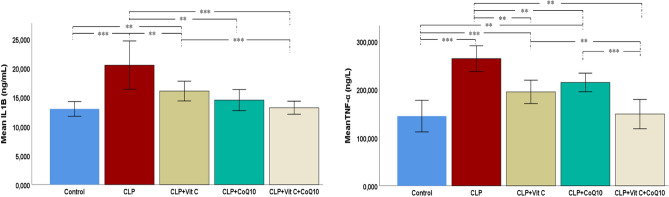
Effects of Vit C, CoQ10 and Vit C + CoQ10 on cardiac IL-1β and TNF-α levels in septic rats (n = 6). Tukey HSD and Games-Howell post-hoc test results used in multiple comparison of group means after one-way ANOVA analysis are shown with *. *p < 0.05, **p < 0.01, ***p < 0.001.

Figure 5 presents the changes in inflammatory biomarkers, specifically IL-1β and TNF-α, across the experimental groups. In the CLP group, there was a significant elevation in both IL-1β and TNF-α levels compared to the control group, reflecting the pronounced inflammatory response induced by sepsis (*p* < 0.001). The introduction of therapeutic agents resulted in reductions in these elevated levels. Treatment with Vit C alone decreased IL-1β and TNF-α, albeit not to control levels. CoQ10 treatment showed a greater anti-inflammatory effect, with a more substantial reduction in IL-1β, closely approaching the control group's level, and a decrease in TNF-α levels. Most significantly, the combined therapy of Vit C and CoQ10 displayed the highest efficacy in moderating these inflammatory markers. This combined treatment brought both IL-1β and TNF-α levels down to near-control values, underlining the synergistic potential of Vit C and CoQ10 in mitigating the inflammatory response in sepsis-induced cardiac injury.

### Evaluation of CK-MB levels post CLP-induced sepsis

**Figure 6:**
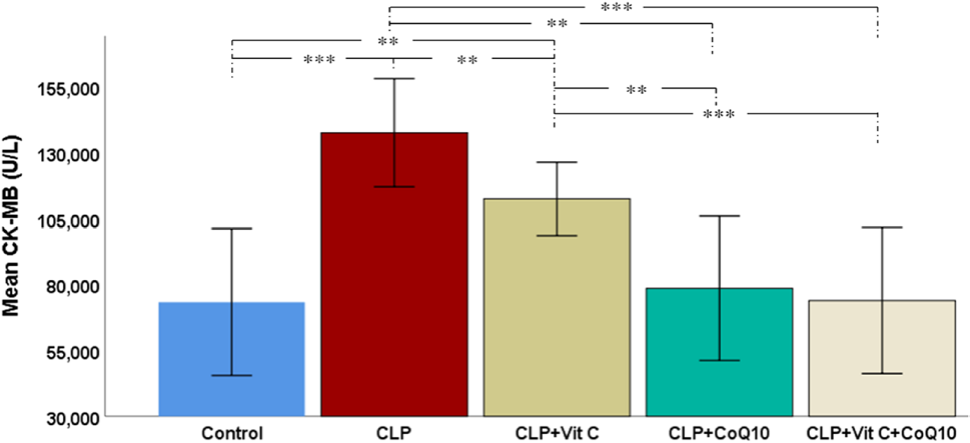
Effects of Vit C, CoQ10 and Vit C + CoQ10 on cardiac CK-MB levels in septic rats (n = 6). Tukey HSD post-hoc test results used in multiple comparison of group means after one-way ANOVA analysis are shown with *. *p < 0.05, **p < 0.01, ***p < 0.001.

As indicated in Fig. 6, following CLP-induced sepsis, significant differences in CK-MB levels, a crucial cardiac biomarker, were observed among the experimental groups. The increase in CK-MB levels in the CLP group compared to the control group highlighted the cardiac impact of sepsis. Upon introducing treatment interventions, a clear trend of amelioration was noted. Vit C treatment led to a reduction in CK-MB levels, suggesting its effectiveness in mitigating cardiac injury due to sepsis. CoQ10 treatment showed an even greater reduction, underscoring its potential in protecting cardiac health under septic conditions. The treatment of Vit C with CoQ10 exhibited the most significant effect, with CK-MB levels.

### Histopathological results

**Figure 7:**
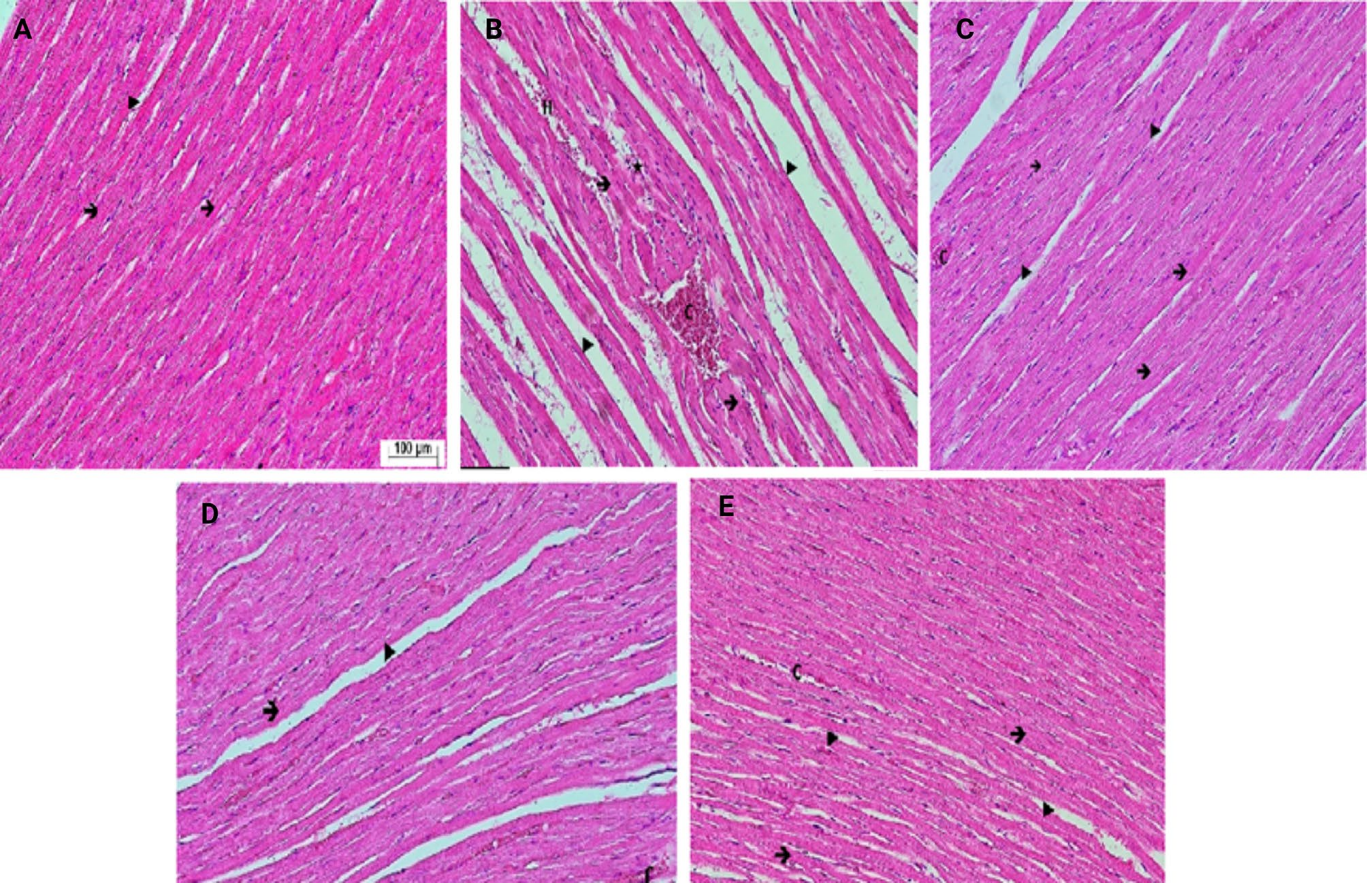
Histopathological evaluations of cardiac tissues post CLP-induced sepsis (Hematoxylin and Eosin Staining, × 200). (**A**) (Control): Heart tissue displays characteristic features with clearly defined cardiac muscle fibers () and intervening spaces (). (**B**) (CLP): Signs of cardiac tissue stress are evident. There are degenerated heart muscle fibers (), interspersed gaps (), along with signs of congestion (**C**), hemorrhage (**H**), and mononuclear cell infiltration (). 7C (Vit C): The heart tissue, post Vit-C treatment, exhibits predominantly preserved cardiac muscle fibers (), with noticeable intervening spaces (). However, mild congestion (**C**) can still be discerned. 7D (CoQ10): Heart tissue CoQ10 treatment demonstrates marked preservation of cardiac muscle fibers () and defined gaps (), with some areas showing congestion (**C**). 7E (Vit C + CoQ10): Following combined treatment, cardiac tissues showcase well-preserved muscle fibers () and intervening spaces (), mild congestion (**C**) remains discernible.

Figure 7 depicts the histopathological results of the study, illustrating the differences in cardiac tissue structure across the groups (H&E). The control group exhibited a normal cardiac structure with well-organized, branched muscle fibers (Fig. 7A). Contrastingly, the CLP group displayed notable irregularities, including degenerated cardiac muscle fibers, enlarged interstitial gaps, vascular congestion, hemorrhagic areas, and mononuclear cellular infiltration, indicating substantial tissue damage due to sepsis (Fig. 7B). In the Vit C group, there was a visible reduction in the gaps between cardiac fibers and lessened areas of congestion, suggesting some mitigation of sepsis-induced damage (Fig. 7C). The CoQ10 group showed further improvement with more orderly arranged cardiac muscle fibers and narrowed interstitial spaces, indicative of significant tissue recovery (Fig. 7D). Notably, the heart tissues in the combined Vit C and CoQ10 group closely resembled the control group, with a regular cardiac structure and minimal signs of sepsis-induced pathology (Fig. 7E).

**Figure 8:**
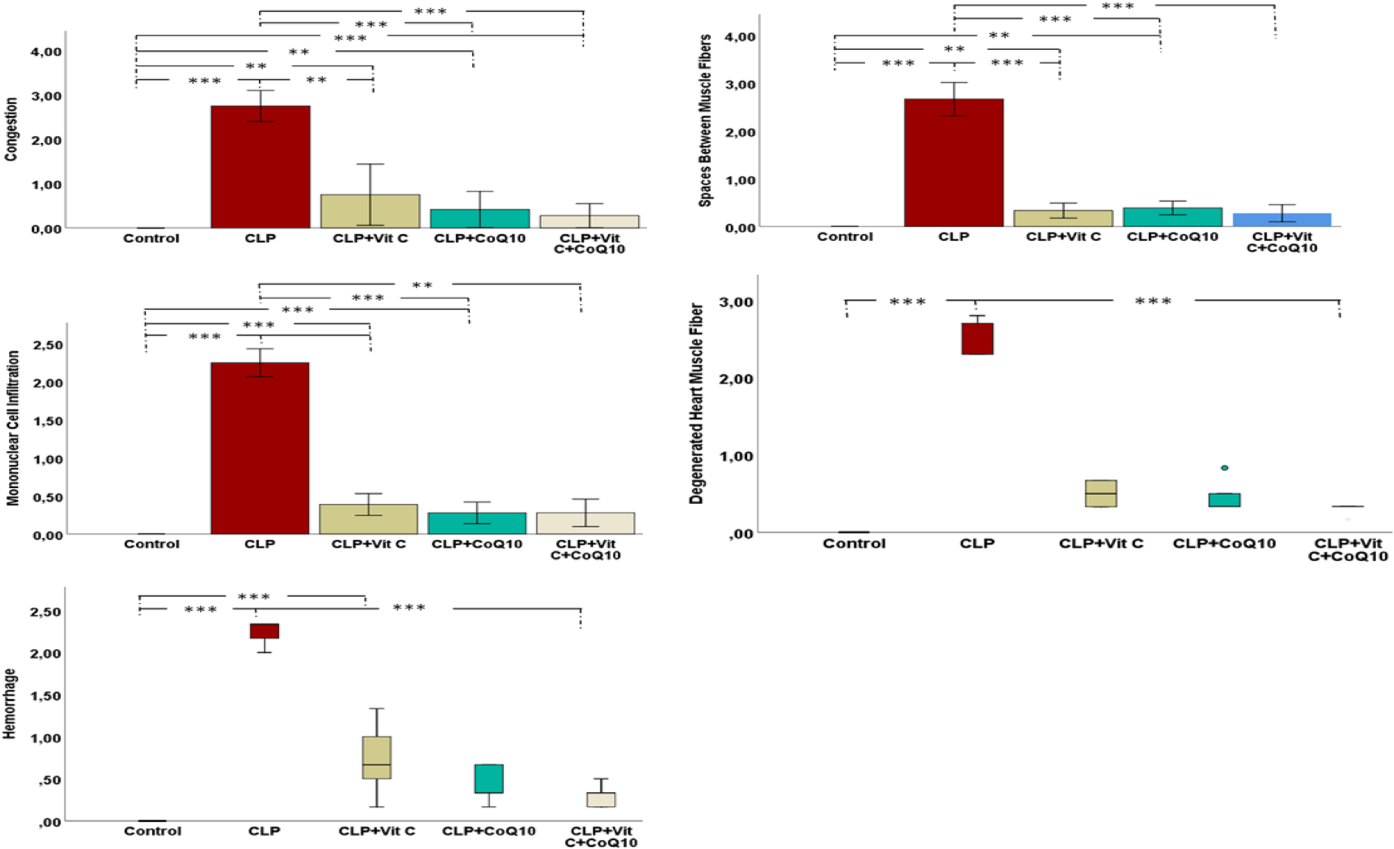
Effects of Vit C, CoQ10 and Vit C + CoQ10 on cardiac histopathological levels in septic rats (n = 6). Games-Howell post-hoc test or Mann–Whitney U test with Bonferroni correction results used in multiple comparison of group means after one-way ANOVA and Kruskal–Wallis analysis are shown with *. *p < 0.05, **p < 0.01, ***p < 0.001.

As illustrated in Fig. 8, a detailed breakdown of the histopathological findings across different groups. The histopathological results provide an in-depth understanding of the structural modifications that sepsis imposes on cardiac tissues and how various treatments, individually or in combination, can potentially reverse or mitigate these alterations. Both Vit C and CoQ10, especially in combination, demonstrate promising effects in preserving the integrity of cardiac tissues post-sepsis.

## Discussion

In the expanding landscape of cardiovascular research, the consequences of sepsis on cardiac tissue and the potential therapeutic interventions to mitigate the deleterious effects have remained paramount concerns. Sepsis, being a life-threatening condition, is often associated with the risk of acute cardiac dysfunction. This study ventured into the promising realm of exploring the protective effects of Vit C, CoQ10, and their combined application against inflammatory heart injury induced by CLP in rat models, mimicking the condition of polymicrobial sepsis.

Historically, Vit C has been studied extensively for its antioxidant properties. Its role in combating oxidative stress and inflammation has been corroborated by several studies in the past^5,21,25^. Similarly, CoQ10, an endogenously produced lipid-soluble component, has shown significant potential in cellular energy production and acts as a strong antioxidant, defending against oxidative damage^41^. What remains a novelty in our study is the exploration of these two compounds' synergistic effect. Combining the distinct mechanisms of action of Vit C and CoQ10 could, theoretically, provide a more comprehensive protective strategy against cardiac damage during sepsis.

Sepsis frequently instigates a profound disturbance in the body's redox homeostasis, especially affecting the delicate cardiac tissues. Initial phases of sepsis are punctuated by a hyper-inflammatory surge, marked by a deluge of inflammatory cytokines like IL-1, IL-6, and notably, TNF-α^42^. TNF-α, as a premier cytokine originating from lipopolysaccharide-stimulated monocytes and macrophages, triggers an extensive inflammatory cascade which can amplify the extent of cardiac injury^43^. Concurrent with this cytokine inundation is the rampant escalation in free radical production, as evidenced by prior studies indicating that heightened TNF-α concentrations directly correlate with increased free radical levels^44^.

Our findings mirrored this perturbation, as we observed an uptick in MDA levels, a terminal product of lipid peroxidation typifying oxidative stress, in sepsis-afflicted cardiac tissues. Noteworthy is the body's inherent reliance on free oxygen radicals as a crucial defense against bacterial invasions^45^. However, during sepsis, this production is overwhelmingly amplified^46^. Historically, increased lipid peroxidation has been recorded in septic patients^47^, in juxtaposition with a decline in cardinal antioxidant enzymes^5^. This escalated oxidant milieu wreaks havoc at the cellular level in cardiac tissues, damaging proteins, lipids, and nucleic acids, culminating in severe endothelial dysfunction, which is especially detrimental in the context of heart health^48,49^.

Given this backdrop, it is hardly surprising that there is a burgeoning interest in devising antioxidant therapeutic strategies, both experimentally and clinically, targeting sepsis. Reinforcing this, a prior study elucidated the surge in MDA, coupled with a concomitant drop in antioxidants like GSH and SOD, in cardiac tissues under the duress of CLP-induced sepsis^50^.

The pathophysiology of sepsis is underpinned by intricate cellular and molecular interactions that drive the inflammatory response. Our study examined the complex interplay of cytokine storms and their pivotal role in exacerbating cardiac injuries during sepsis. Specifically, the uncontrolled release of pro-inflammatory cytokines such as IL-1β, IL-6, and TNF-α leads to myocardial depression, endothelial dysfunction, and triggers apoptosis pathways in cardiac cells^51^.

Our results provide compelling evidence that the combined administration of Vit C and CoQ10 offers a significant protective shield against this inflammation-induced cardiac derangement. The synergy between these agents appeared to target multiple facets of the inflammation cascade. Vit C, a potent antioxidant, is known to inhibit the transcription factor NF-kB, which orchestrates the expression of various pro-inflammatory genes^52^. Simultaneously, CoQ10, an essential component of the mitochondrial electron transport chain, not only supports ATP synthesis but also modulates inflammation by inhibiting the release of pro-inflammatory cytokines and by down-regulating the NLRP3 inflammasome^53^. These findings, in tandem with our data, underscore the potential therapeutic advantage of dual supplementation with Vit C and CoQ10 in septic cardiac complications. Their combined effect seems to dampen the severity of the cytokine storm and, in turn, mitigate its detrimental impacts on the heart.

NO plays a multifaceted role in the pathophysiology of sepsis, acting as a double-edged sword. While physiological concentrations of NO are essential for various cellular functions, its overproduction, particularly from the upregulation of inducible nitric oxide synthase (iNOS), can have deleterious effects in the context of sepsis^54,55^. High levels of NO, in conjunction with ROS, can lead to the formation of peroxynitrite, a potent oxidant that can induce lipid peroxidation, DNA damage, and protein modification^56^. Moreover, excessive NO can cause vasodilation, leading to hypotension, one of the defining features of septic shock^57^. In septic conditions, the unregulated production of NO via iNOS is believed to mediate the suppression of cardiac contractility, leading to myocardial dysfunction^58^. Furthermore, there's evidence suggesting that iNOS-derived NO contributes to mitochondrial dysfunction in cardiomyocytes, thereby exacerbating energy depletion in an already stressed environment^59^. Our findings hint that the combined application of Vit C and CoQ10 can potentially attenuate the overproduction of NO, possibly by modulating iNOS expression or activity. However, the intricate molecular mechanisms underlying this observation, whether through direct interaction with iNOS or by counteracting the downstream effects of excessive NO, remain to be elucidated in detail.

Our results clearly highlighted the benefits of these therapeutic interventions. Histopathological evaluations underscored that while sepsis led to degenerated heart muscle fibers, expanded spaces between the fibers, congestions, hemorrhagic areas, and notable mononuclear cellular infiltration, these effects were significantly mitigated by Vit C, CoQ10, and especially their combined administration. The combined treatment regime showed a histopathology almost similar to the sham control group, indicating a near-complete reversal of the injurious effects of sepsis.

Given the results observed in our study, future research should focus on further elucidating the mechanisms underlying the synergistic effects of Vit C and CoQ10 in sepsis-induced cardiac dysfunction. Long-term studies are needed to assess the sustainability and safety of these treatments over extended periods. Additionally, exploring the effectiveness of these interventions in different models of sepsis and varying severity levels could provide more comprehensive insights. Clinical trials are also imperative to translate these findings into practical therapeutic strategies for human patients suffering from sepsis. Furthermore, investigating the potential of these agents in other organ systems affected by sepsis could broaden the scope of their therapeutic applications. Our study lays the groundwork for these future avenues of research, potentially leading to more effective and multifaceted treatment strategies against the complex challenges posed by sepsis.

## Conclusion

This study unveiled, for the first time, the potent synergistic effects of combined Vit C and CoQ10 administration in counteracting inflammatory cardiac injury in a CLP-induced polymicrobial sepsis model in rats. Interestingly, in certain aspects, CoQ10 displayed superior efficacy compared to Vit C, suggesting its critical role in the combined therapeutic approach. Our findings underscore the potential of these compounds in mitigating the oxidative and inflammatory cascades, as well as in modulating nitric oxide dynamics, pivotal in sepsis pathophysiology. The implications of this study indicating a promising therapeutic strategy for managing cardiac complications in septic patients. Further studies are crucial to decode the precise molecular mechanisms and assess the clinical applicability of this combined therapy in diverse patient populations.

## Data Availability

The datasets used and/or analyzed during the current study are available from the corresponding author on reasonable request.
